# Supplementary material for: Content-rich biological network constructed by mining PubMed abstracts
Source: BMC Bioinformatics. 2004 Oct 8;5:147. doi: 10.1186/1471-2105-5-147 (PMC528731; doi:10.1186/1471-2105-5-147)
Supplement: Additional File 2 — The original results of the above study (non-essential files are deleted to keep the file size under the limit set by BMC bioinformatics). [file 1471-2105-5-147-S2.bz2 › chilibotAdditionalFile2/dip20/187ID7644522E666/html/ECR_USP.html]

 


 **ECR** and **USP** 
  
Found 116 abstracts in PubMed, retrieved 20.  
 

 What does Google say? 
 PDF only 
| .edu only 

---

**Interactive relationship** (e.g. stimulation, inhibition, etc)

**Neutral relationship**- Immunoprecipitation assays demonstrated that both the p60 and p64  **USP**  coprecipitated with the p65  **EcR** .  Ref: 12878229 Insect Biochem Mol Biol, 2003
- **USP**  acts as an allosteric effector for  **EcR** , but does not contribute any intrinsic function.  Ref: 12554759 Mol Endocrinol, 2003
- The insect ecdysteroid receptor  [ **ECR** ]  consists of a heterodimer between  **EcR**  and the RXR orthologue,  **USP** .  Ref: 12084064 Eur J Biochem, 2002
- The ecdysone receptor  [ **ECR** ]  is a heterodimer of the two nuclear receptors  **EcR**  and ultraspiracle  [ **USP** ]   **USP** .  Ref: 12554759 Mol Endocrinol, 2003
- The functional ecdysone receptor  [ **ECR** ]  is a heterodimer of  **ECR**  NR1H1 and  **USP**  RXR NR2B4, which is the orthologue of vertebrate retinoid X receptors RXR alpha, beta, gamma.  Ref: 12654933 Mol Biol Evol, 2003
- In the ultraspiracle  [ **USP** ]   **Usp**  protein, which together with  **EcR**  forms the heterodimeric ecdysone receptor  [ **ECR** ] , the P box residues are E19, G20 and G23.  Ref: 12422244 Acta Biochim Pol, 2002
- The functional ecdysone receptor  [ **ECR** ]  is a heterodimer composed of the ecdysone receptor  [ **ECR** ]   **EcR**  and ultraspiracle  [ **USP** ] , the homolog of the retinoid X receptor.  Ref: 12370123 J Mol Endocrinol, 2002
- In insect, only the ecdysone receptor  [ **ECR** ]  and hormone receptor 38 are known thus far to dimerize with  **USP** .  Ref: 12039078 Mol Cell Endocrinol, 2002
- Our data provide the first evidence that  **ECR**  and  **USP**  RXR may have coevolved during holometabolous insect diversification, leading to a functional divergence of the ecdysone receptor  [ **ECR** ] .  Ref: 12654933 Mol Biol Evol, 2003
- Western blot analysis revealed that the expression of  **EcR**  and  **USP**  was significantly increased in tebufenozide and methoxyfenozide treated samples compared to the control, suggesting that ecdysone agonists regulated the Vg synthesis via the  **EcR**  and  **USP**  proteins complex.  Ref: 12587140 Arch Insect Biochem Physiol, 2003

**Non-interactive relationship** (e.g. studied together, co-existance, homology, etc.)

- In the present study, the effects of these ecdysone agonists, tebufenozide and methoxyfenozide, on the expression of ecdysone receptor  [ **ECR** ]   **EcR** , ultraspiracle  [ **USP** ]  protein  **USP**  and other proteins in the ovaries of C. pomonella L.  Ref: 12878229 Insect Biochem Mol Biol, 2003
- These hormonal responses are thought to be mediated by the ecdysteroid receptor  [ **ECR** ]   **EcR**  and its heterodimeric partner Ultraspiracle  [ **USP** ]   **USP** .  Ref: 12809604 Cell, 2003
- This gene encodes the BR C Z1 transcription factor and its expression is directly regulated by ecdysone through the ecdysone receptor  [ **ECR** ]   **EcR**   **Usp** .  Ref: 12490155 J Struct Biol, 2002
- We have identified the regions of Drosophila  **EcR**  and  **USP**  responsible for transcriptional activation of a semisynthetic Eip71CD promoter in Kc cells.  Ref: 12554759 Mol Endocrinol, 2003
- In gel mobility shift assays, in vitro translated C. suppressalis  **EcR**  B1 CsEcR B1 and Bombyx mori ultraspiracle  [ **USP** ]  BmUSP proteins bound to the Pal 1 and Drosophila melanogaster hsp27 ecdysone response element as a heterodimer.  Ref: 12213236 Insect Biochem Mol Biol, 2002
- Two members of the nuclear receptor superfamily,  **EcR**  ecdysteroid receptor  [ **ECR** ]  protein and  **Usp**  Ultraspiracle  [ **USP** ] , heterodimerize to form a functional receptor for the steroid hormone 20 hydroxyecdysone and thus enable it to coordinate morphogenetic events during insect metamorphosis.  Ref: 12674500 Biol Chem, 2003
- Moreover, a highly synergistically formed heterodimeric complex with the DNA binding domain of  **EcR**  was observed on hsp27EcRE, but not on the native  **Usp**  response element from the chorion s15 gene promoter.  Ref: 12674500 Biol Chem, 2003
- Many of these genes are transcriptionally regulated by the steroid hormone ecdysone and play a role during the onset of metamorphosis, including the  **EcR**   **USP**  ecdysone receptor  [ **ECR** ]  heterodimer.  Ref: 12881508 Mol EndocrinolMol Endocrinol, 2003
- Overall, 38  **USP**  RXR and 19  **ECR**  protein sequences, from 33 species, have been used for this analysis.  Ref: 12654933 Mol Biol Evol, 2003
- Effects of ecdysone agonists on the expression of  **EcR** ,  **USP**  and other specific proteins in the ovaries of the codling moth Cydia pomonella L.  Ref: 12878229 Insect Biochem Mol Biol, 2003
- The data suggest that the ecdysone agonists regulate, via the  **EcR**   **USP**  complex, the expression of these specific proteins that might eventually lead to the inhibition of fecundity in the codling moth.  Ref: 12878229 Insect Biochem Mol Biol, 2003
- Transcription Activation by the Ecdysone Receptor  [ **ECR** ]   **EcR**   **USP**  Identification of Activation Functions.  Ref: 12554759 Mol Endocrinol, 2003
- In order to gain further insight into the evolution of metamorphosis and gene regulation by ecdysone in arthropods, we performed a phylogenetic analysis of both partners of the heterodimer  **ECR**   **USP**  RXR.  Ref: 12654933 Mol Biol Evol, 2003
- DHR38 also heterodimerizes with  **USP** , and this complex responds to a distinct class of ecdysteroids in a manner that is independent of  **EcR** .  Ref: 12809604 Cell, 2003
- p64 was a dominant  **USP**  to form complex with  **EcR** .  Ref: 12878229 Insect Biochem Mol Biol, 2003
- Ligand induced heterodimerization between the ligand binding domains of the Drosophila ecdysteroid receptor  [ **ECR** ]  and ultraspiracle  [ **USP** ] .  Ref: 12084064 Eur J Biochem, 2002
- The effects of tebufenozide and methoxyfenozide on vitellogenin Vg synthesis release in the fat body, translocation in hemolymph, uptake by the ovary, and the expression of the ecdysone receptor  [ **ECR** ]   **EcR**  and its heterodimer partner, ultraspiracle  [ **USP** ]  protein  **USP**  in fat body, were investigated in Cydia pomonella.  Ref: 12587140 Arch Insect Biochem Physiol, 2003
- Mutagenesis reveals that AF2 function depends on  **EcR**  helix 12, but not on the cognate  **USP**  region.  Ref: 12554759 Mol Endocrinol, 2003
- Interestingly, sequence alignments and structural comparisons reveal high divergence rates, for both  **ECR**  and  **USP**  RXR, specifically among Diptera and Lepidoptera.  Ref: 12654933 Mol Biol Evol, 2003
- The isoform specific A B domains of  **EcR**  B1 and B2, but not those of  **EcR**  A or  **USP** , exhibit strong activation activity activation function 1 AF1, both in isolation and in the context of the intact receptor.  Ref: 12554759 Mol Endocrinol, 2003
- Effect of ecdysone agonists on vitellogenesis and the expression of  **EcR**  and  **USP**  in codling moth Cydia pomonella.  Ref: 12587140 Arch Insect Biochem Physiol, 2003
- Rapid Divergence of the Ecdysone Receptor  [ **ECR** ]  in Diptera and Lepidoptera Suggests Coevolution Between  **ECR**  and  **USP**  RXR.  Ref: 12654933 Mol Biol Evol, 2003
- To develop an ecdysone receptor  [ **ECR** ]   **EcR**  based inducible gene regulation system, several constructs were prepared by fusing DEF domains of Choristoneura fumiferana  **EcR**  CfEcR, C. fumiferana ultraspiracle  [ **USP** ]  CfUSP, Mus musculus retinoid X receptor MmRXR to either GAL4 DNA binding domain DBD or VP16 activation domain.  Ref: 12631289 Eur J Biochem, 2003
- Mutants created by site directed mutagenesis were used to elucidate the function of amino acids involved in ligand binding to ecdysteroid receptor  [ **ECR** ]   **EcR**  and heterodimer formation with ultraspiracle  [ **USP** ]   **USP** .  Ref: 12674504 Biol Chem, 2003
- Therefore, ecdysteroids are capable of inducing heterodimer formation between  **EcR**  and  **USP** , even when the binding of these receptor proteins to cognate DNA response elements does not occur.  Ref: 12084064 Eur J Biochem, 2002
- The absence of in vivo effects due to these steroid agonists and antagonists prompted in vitro studies of ecdysteroid receptor  [ **ECR** ]  activity of a range of reference compounds and environmental contaminants with an ecdysteroid receptor  [ **ECR** ]   **EcR**   **USP**  based screening assay derived from the BII haemocyte line of Drosophila melanogaster.  Ref: 12408640 Mar Environ Res< MedlineTA>Mar Environ Res, 2002
- We found that C terminal protein fragments that comprised the ligand binding, but not the DNA binding domain of  **EcR**  and  **USP**  and which were equipped with the activation or DNA binding region of GAL4, respectively, exhibit a weak ability to interact spontaneously with each other.  Ref: 12084064 Eur J Biochem, 2002
- Different effects of the same point mutations on ligand binding to  **EcR**  and  **EcR**   **USP**  R511, A612, L615, I617, T619, N626 indicate that the ligand binding pocket is modified by heterodimerization.  Ref: 12674504 Biol Chem, 2003
- Western and Northern blot analyses revealed that the expression of a 65 kDa p65  **EcR** , and 60 and 64 kDa p60 and p64  **USP**  proteins were enhanced by tebufenozide and methoxyfenozide at both transcriptional and translational levels.  Ref: 12878229 Insect Biochem Mol Biol, 2003
- Here, we report that mosquito Seven up AaSvp, a chicken ovalbumin upstream promoter transcription factor homologue, is involved in regulating the cyclicity of vitellogenic ecdysteroid mediated signaling through heterodimerization with a retinoid X receptor homologue Ultraspiracle  [ **USP** ]   **USP** , the obligatory functional ecdysteroid receptor  [ **ECR** ]   **EcR**  partner.  Ref: 12522263 Proc Natl Acad Sci U S A, 2003
- The proximal region, containing binding sites to  **EcR**   **USP** , GATA, C EBP and HNF3 fkh, is required for the correct tissue and stage specific expression at a low level.  Ref: 12225918 Insect Biochem Mol Biol, 2002
- AF2 function was studied using a two hybrid assay in Kc cells, based on the observation that potent hormone dependent activation by the  **EcR**   **USP**  ligand binding domain heterodimer requires the participation of both partners.  Ref: 12554759 Mol Endocrinol, 2003
